# Microbial diversity of garden snail mucus

**DOI:** 10.1002/mbo3.1263

**Published:** 2022-02-08

**Authors:** Mihaela Belouhova, Elmira Daskalova, Ivaylo Yotinov, Yana Topalova, Lyudmila Velkova, Aleksander Dolashki, Pavlina Dolashka

**Affiliations:** ^1^ Faculty of Biology Sofia University “St. Kliment Ohridski” Sofia Bulgaria; ^2^ Institute of Organic Chemistry with Centre of Phytochemistry Bulgarian Academy of Sciences Sofia Bulgaria

**Keywords:** *Acinetobacter*, *Cornu aspersum*, *Pedobacter*, *Rhizobiaceae*, snail mucus

## Abstract

The search for new natural compounds for application in medicine and cosmetics is a trend in biotechnology. One of the sources of such active compounds is the snail mucus. Snail physiology and the biological activity of their fluids (especially the mucus) are still poorly studied. Only a few previous studies explored the relationship between snails and their microbiome. The present study was focused on the biodiversity of the snail mucus used in the creation of cosmetic products, therapeutics, and nutraceuticals. The commonly used cultivation techniques were applied for the determination of the number of major bacterial groups. Fluorescence in situ hybridization for key taxa was performed. The obtained images were subjected to digital image analysis. Sequencing of the 16S rRNA gene was also done. The results showed that the mucus harbors a rich bacterial community (10.78 × 10^10^ CFU/ml). Among the dominant bacteria, some are known for their ability to metabolize complex polysaccharides or are usually found in soil and plants (*Rhizobiaceae*, *Shewanella*, *Pedobacter*, *Acinetobacter*, *Alcaligenes*). The obtained data demonstrated that the snail mucus creates a unique environment for the development of the microbial community that differs from other parts of the animal and which resulted from the combined contribution of the microbiomes derived from the soil, plants, and the snails.

## Graphical abstract

Bacteria *Acinetobacter sp. and the Rhizobiaceae family, (Pedobacter sp., Aeromonas sp., Shewanella sp., and Alcaligenes sp.)* were identified as main members of the snail mucus‐associated bacterial community.

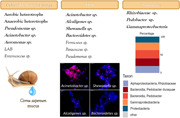

## 1 INTRODUCTION

The mollusks belong to invertebrates and have enormous diversity (100–200 thousand species). They comprise approximately 7% of living animals (Ahmad et al., 2018; Benkendorff, 2010). The biodiversity that characterizes them is reflected by their chemical composition and the production of varieties of secondary metabolites (Ahmad et al., 2018). Mollusks do not possess acquired immunity. At the same time, they live in an environment that contains diverse microorganisms, and are exposed to many pathogens (Eghianruwa et al., 2019). The molluscan defense mechanisms are based on innate immunity and the presence of secondary metabolites. A distinguishable class of them are the ones with antibacterial and antiviral activity (Babar et al., 2012; Dang et al., 2015; Dolashki et al., 2020). From the organisms in question metabolites with many other activities are isolated—anti‐cancer (Edwards et al., 2012; Jo et al., 2017; Suarez‐Jimenez et al., 2012), antioxidant (Pangestuti & Kim, 2017; Qian et al., 2008), wound healing (Badiu et al., 2010; Benkendorff et al., 2015; Chen et al., 2016), anticoagulant (Gomes et al., 2010; Jung & Kim, 2009; Mohan et al., 2016; Vijayabaskar & Somasundaram, 2009; Volpi & Maccari, 2005). Because of their diverse beneficial effects, the mollusks are used in medicine worldwide (Ahmad et al., 2018).

Different parts of the snail have different compositions of their biologically active substances. For example, several studies focused on shell (Chen et al., 2016; Latire et al., 2014), body (Iijima et al., 2003; Li et al., 2014), mucus (Dolashki et al., 2020; Greistorfer et al., 2017; Zhong et al., 2013; Ziomek et al., 2017), and hemolymph (Dharmu et al., 2007; Dolashka et al., 2011; Sperstad et al., 2011). Marine snails (Jo et al., 2017; Khan & Liu, 2019), as well as terrestrial ones (Chinaka et al., 2021; Pitt et al., 2015) are used for extraction of active substances.

On the other hand, it is already clear that the macroorganisms are modified and functionally complemented by their microbiome. This is also important in regards to several aspects concerning the snails—for clarification of the role of the symbiotic bacteria in the synthesis and degradation of the molluscan active substances; in maintaining the homeostasis; in the building of barrier against infections with pathogenic bacteria. Only a few studies could be found on the matter. Silva et al. (2013) report for the dominating role of *Aeromonas, Citrobacter, Enterobacter, Cupriavidus, Rhizobium, Stenotrophomonas, Pseudomonas, Klebsiella, Acinetobacter, Vibrio*, and *Sphingomonas* in the microbiome of *Biomphalaria glabrata*. Dominating species in free‐living representatives of the same snails is *Enterobacter cloacae*, while in the lab‐grown animals *Citrobacter freundii* and *Aeromonas sobria* are found in the highest numbers. Charrier and coworkers demonstrated that the gut microbiome of the garden snails *Cornu aspersum* and *Helix pomatia* is constituted mainly of *Gammaproteobacteria* and *Firmicutes* (Charrier et al., 2006). Simkiss established a bacterial count of 0.71 × 10^6^ CFU/g of body weight of snails *C. aspersum* (Simkiss, 1985).

Although the snail mucus is used in traditional medicine as well as in innovative natural products for the treatment of different health problems, there is no information about this snail fluid microbiome. That is why this study aims to give information about the microbial diversity of snail mucus used in cosmetic products and food supplements. An investigation of the mucus chemical composition using mass spectrometry was performed. Several genetic and cultivation methods that can give complementary information were used for the determination of the main bacterial groups in *C. aspersum* mucus. The study focused on microbial groups that are widely spread in the environment, because of the close contact of the snails with the surroundings.

## 2 MATERIALS AND METHODS

### 2.1 Mucus collection from snails *Cornu aspersum* and preparation of extract

*C. aspersum* snails were grown in Bulgarian eco‐farms that cultivate them for medicinal and nutritional use. The mucus was collected from the foot of 100 snails without disruption of their biological functions. After washing with distilled water, these snails were placed in a special device where they secrete mucus after electrical stimulation with a low voltage current. The mucus thus obtained was homogenized and subjected to centrifugation at ×3000 g to remove coarse impurities. The supernatant was subjected to several filtration cycles using filters with smaller pore sizes for each subsequent filtration. After several stages of homogenization and purification, an extract is obtained which is used for analysis.

**Table 1:** Oligonucleotide probes used in the fluorescence in situ hybridization (FISH) experiments

No.	Microorganisms	Probe sequence	Fluorescent dye	Formamide concentration	References
1	*Acinetobacter* sp.	5′‐ATC CTC TCC CAT ACT CTA‐3′	Cy3	35%	Wagner et al. (1994)
2	*Alcaligenes* sp.	5′‐CCG AAC CGC CTG CGC AC‐3′	Cy3	35%	Friedrich et al. (2003)
3	*Shewanella* sp.	5′‐AGC TAA TCC CAC CTA GGT WCA TC‐3′	Cy3	40%	Loy et al. (2007)
4	*Bacteroidetes* sp.	5′‐AGC TGC CTT CGC AAT CGG‐3′	Cy3	30%	Weller et al. (2000)
5	*Firmicutes* sp.	5′‐TGG AAG ATT CCC TAC TGC‐3′	Cy3	20%	Meier et al. (1999)
		5′‐CGG AAG ATT CCC TAC TGC‐3′			
		5′‐CCG AAG ATT CCC TAC TGC‐3′			
6	*Paracoccus* sp.	5′‐GGA TTA ACC CAC TGT CAC C‐3′	Cy3	20%	Neef et al. (1996)
7	*Pseudomonas* sp.	5′‐GCT GGC CTA GCC TTC‐3′	Cy3	20%	Schleifer et al. (1992)
8	*NON‐EUB*	5′‐ACT CCT ACG GGA GGC AGC‐3′	Cy3	20–40%	Wallner et al. (1993)

### 2.2 Microbiological analyses

Plate count techniques were used to study some of the main bacterial groups from the environment. The aerobic and anaerobic heterotrophs were cultivated on nutrient agar (HiMedia). The aerobic heterotrophs were incubated for 48 h while the anaerobic ones—for 7 days in an anaerobic environment (Anaerocult, Merck). The lactic acid bacteria (LAB) were cultivated on MRS agar (HiMedia) for 7 days in anaerobic conditions (Anaerocult, Merck). The other studied bacteria were *Pseudomonas* sp. (GSP agar, HiMedia), *Aeromonas* sp. (GSP agar, HiMedia), *Acinetobacter* sp. (Sellers agar, HiMedia), and *Enterococcus* sp. (Barnes agar, HiMedia).

### 2.3 Fish

Fluorescence in situ hybridization (FISH) was also performed. The samples were fixed according to the protocol by Amann et al. (1995). The FISH analysis was performed according to the protocol of Nielsen et al. (2009). The abundance of seven bacterial groups was investigated: *Acinetobacter* sp., *Alcaligenes* sp., *Shewanella* sp., *Bacteroidetes* sp., *Firmicutes* sp., *Paracoccus* sp., *Pseudomonas* sp. The oligonucleotide probes were chosen from the database probeBase (Loy et al., 2007) and are described in Table 1. The probe NON‐EUB was used as a negative control. For quantification of the target bacteria, the software *daime* was used (Daims et al., 2006). The threshold criteria for segmentation of the FISH images were chosen manually. The results are represented as a percentage of the target bacteria of all bacteria (stained by DAPI). The pictures were taken on an epifluorescent microscope Leica DMB6.

### 2.4 Sequencing

In the study, metagenomic sequencing of the 16S rRNA gene was also performed. The bacteria in the mucus were concentrated from 80 ml samples by centrifugation at 4000 rpm. The pellet was washed three times with PBS for eliminating any interference from inhibitors in the mucus. For extraction of the genomic DNA, the Zymo Quick‐DNA Fungal/Bacterial Miniprep Kit (Zymo Research, D6005) was used. Only samples with DNA concentrations above 7 ng/µl were sent for sequencing. The DNA concentration was determined with SpectraMax QuickDrop Micro Volume Spectrophotometer (Molecular Devices, LLC).

16S rRNA gene amplicon sequencing and bioinformatic analysis of the sequencing data was performed by omics2view.consulting GbR. Fusion primers (containing P5/P7 Illumina adapter sequence, 8‐nt index sequence, and the gene‐specific primer sequence) were used for the preparation of the 16S V3‐V4 libraries (Table 2). The purification of the libraries was performed with Agencourt AMPure XP beads and validated with an Agilent Technologies 2100 bioanalyzer. Sequencing was done on Illumina HiSeq 2500 (2 × 300 bp). Demultiplexing of the raw data was done. The amplicon sequence variants (ASVs) determination was performed with the DADA2 pipeline (Callahan et al., 2016). BBTools package v38.45 was used for removal of the primer sequences within an edit distance of 3 from 5′ and 3′ ends of the input read pairs (Bushnell, 2019). The reads with ≤2 expected errors for the forward direction and with ≤4 expected errors for the reverse direction were retained. Removal of the chimeric contigs was done according to the DADA2 procedure. The IDTAXA approach was applied for the classification of the remaining contigs (ASVs) (Murali et al., 2018). For this, the R package DECIPHER v2.18.1 (Wright, 2016) was used. The analysis was based on GTDB database release 202 (Parks et al., 2018, 2020). The ASVs with a classification confidence value ≥51% were retained. For the construction of a neighbor‐joining phylogenetic tree (Saitou & Nei, 1987) the R package DECIPHER was applied.

**Table 2:** Gene‐specific primer sequences

Primer name	Sequence
341F	ACTCCTACGGGAGGCAGCAG
806R	GGACTACHVGGGTWTCTAAT

The information for the taxonomic composition of the microbial community was obtained from OTU (operational taxonomic unit) counts at the species level. The data is presented at the class level with a grouping of taxa. Taxa with a mean frequency <0.5% across samples are summarized as “other.”

### 2.5 Mass spectrometric analysis of mucus extract

The microbiological analyses were supplemented with mass spectrometric study of the mucus for detection of important compounds in it to elucidate some of the features of the media in which the bacteria in the mucus naturally functioned. The molecular masses of peptides were determined by MALDI‐TOF mass spectrometry on an AutoflexTM III High‐Performance MALDI‐ TOF/TOF System (Bruker Daltonics). The mixture of 2.0 μl of matrix solution (7 mg/ml of α‐cyano‐4‐hydroxycinnamic acid in 50% ACN containing 0.1% TFA) and 2.0 μl of the sample was spotted on a stainless steel target plate. The mixture of angiotensin I, Glu1‐fibrinopeptide B, ACTH (1–17), and ACTH was used for calibration of the mass spectrometer. The MS/MS spectra were recorded in reflector mode and the amino acid sequences (AASs) of peptides were identified by precursor ion fragmentation using MALDI‐MS/MS analysis.

### 2.6 Further analyses

Isoelectric point (pI), grand average of hydropathicity index (GRAVY), and net charge of peptides were determined by ExPASy MW/pI and ExPASy ProtParam (https://web.expasy.org/compute_pi/). The antimicrobial properties of peptides (shown in Table 3) were predicted by iAMPpred‐software (https://cabgrid.res.in:8080/amppred/), based on the identified primary structures using an extensive database. The tool processes several important features such as the AASs, physico‐chemical, and other structural features of the peptides, and identifies the best candidate AMP prior to in vitro experimentation (Meher et al., 2017).

**Table 3:** Characterization of peptides in the mucus from garden snail *Cornu aspersum*, identified by *de novo* MALDI‐MS/MS sequencing

No	Amino acid sequence of peptides	Exper. MW [M + H]^+^ (Da)	Calcul. monoisotopic mass (Da)	pI	GRAVY	Predicted activity		
						Antibacterial (%)	Antiviral (%)	Antifungal (%)
1^a^	LGHDVH	677.33	676.33	5.97	−0.383	84.0	75.8	61.0
2^a^	AAGLAGAGNGGG	872.42	871.41	5.57	+0.425	65.0	35.0	35.0
3	LLFSGGQFNG	1039.52	1038.51	5.52	+0.420	74.0	28.0	59.0
4^a^	LGLGNGGAGGGLVGG	1155.61	1154.60	5.52	+0.687	86.0	50.8	61.0
5^b^	LNLGLDAGGGDPGG	1212.57	1211.58	3.56	−0.093	57.0	59.5	37.6
6	GAACNLEDGSCLGV	1308.81	1307.55	3.67	+0.564	58.0	58.0	53.0
7^b^	NLVGGLSGGGRGGAPGG	1382.70	1381.71	9.75	−0.024	59.0	30.4	38.0
8^b^	LGGLGGGGAGGGGLVGEPG	1438.86	1437.72	4.00	+0.439	56.0	38.0	19.7
9^c^	NLVGGLSGGGRGGAPGGGG	1496.89	1495.75	9.75	−0.063	68.0	40.0	47.0
10^b^	GLLGGGGGAGGGGLVGGLLNG	1609.94	1608.86	5.52	+0.776	90.0	53.6	65.0
11^b^	MGGLLGGVNGGGKGGGGPGAP	1666.83	1665.83	8.5	+0.005	78.6	52.0	61.5
12^d^	LFGGHQGGGLVGGLWRK	1738.99	1737.94	11.0	−0.024	75.6	41.0	78.5
13	NGLFGGLGGGGHGGGGKGPGEGGG	1909.90	1908.88	6.75	−0.487	90.0	67.0	80.0
14	LLLLMLGGGLVGGLLGGGGKGGG	1966.24	1965.14	8.75	+1.209	92.0	57.0	76.0
15	PFLLGVGGLLGGSVGGGGGGGGAPL	2023.14	2022.09	5.96	+0.912	69.0	32.0	38.0
16^b^	LPFLGLVGGLLGGSVGGGGGGGGPAL	2136.20	2135.17	5.52	+1.023	69.1	32.0	38.2
17^b^	DVESLPVGGLGGGGGGAGGGGLVGGNLGGGAG	2479.20	2478.21	3.67	+0.353	62.0	43.0	33.0

Abbreviations: GRAVY, grand average of hydropathicity index; pI, Isoelectric point.

^a^ Amino acid sequences of a peptide is published in Vassilev et al. (2020).

^b^ Amino acid sequences of a peptide is published in Dolashki et al. (2020).

^c^ Amino acid sequences of a peptide is published in Dolashki et al. (2018).

^d^ Amino acid sequences of a peptide is published in Velkova et al. (2018).

## 3 RESULTS

In the mucus from *C. aspersum*, primary structures of nine new peptides with molecular weights between 1000 and 3000 Da were identified by tandem mass spectrometry, and their antibacterial activity was previously reported (Velkova et al., 2018). Metabolic analysis of snail mucus by NMR showed metabolites with known antioxidant, antibacterial, and antimicrobial activity in two low molecular weight fractions (Mw < 1 kDa and Mw < 3 kDa). Some of them were confirmed by tandem mass spectrometry (Table 3).

**Figure 1:**
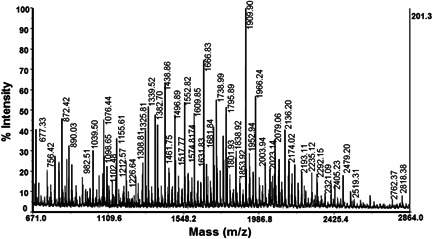
MALDI‐MS spectrum of peptides with Mw below 3 kDa in *Cornu aspersum* mucus by AutoflexTM III, high performance MALDI‐TOF&TOF/TOF Systems (Bruker Daltonics)

**Figure 2:**
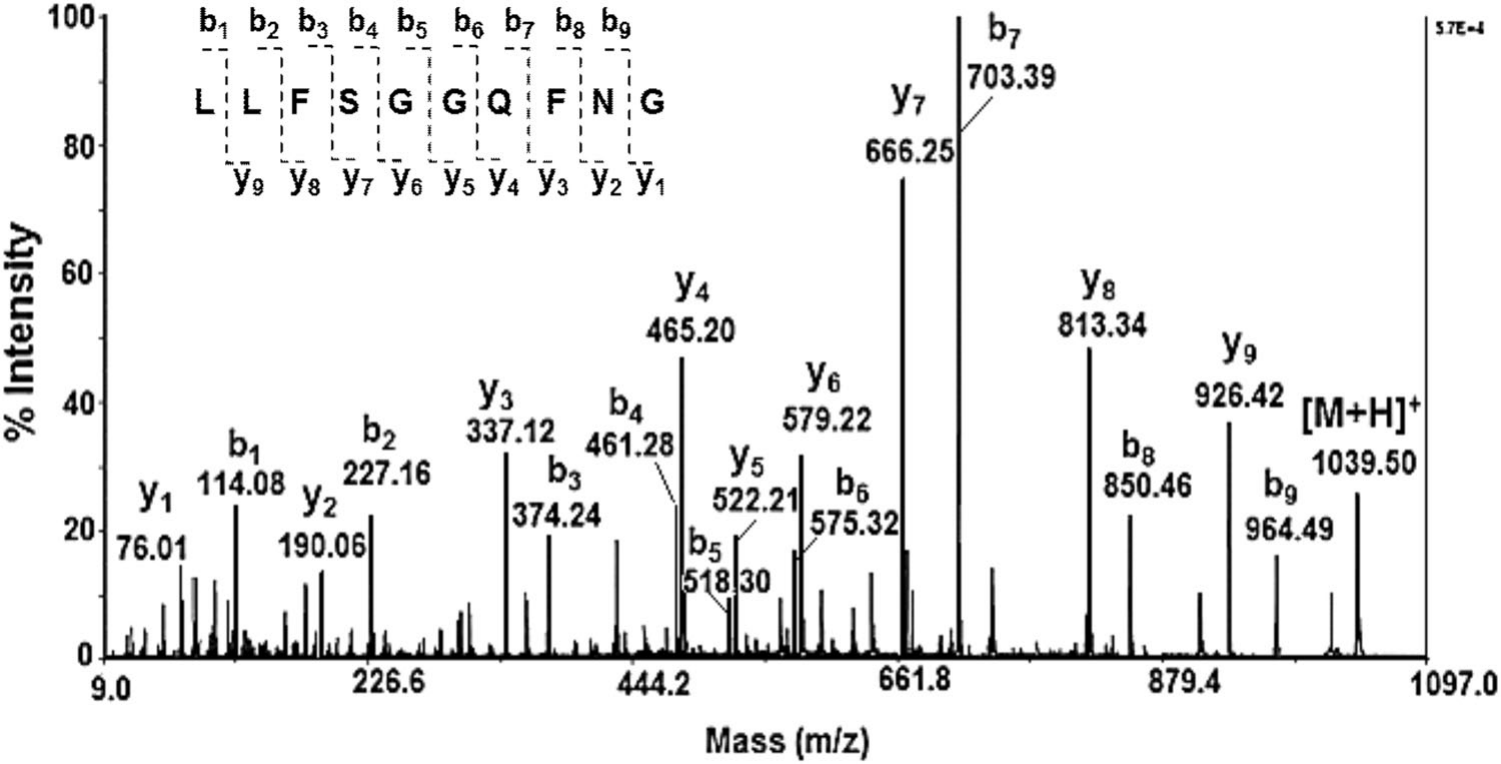
MALDI‐MS/MS analysis of peptide, presented as [M + H]^+^ at m/z 1039.50 Da, with fragmentation nomenclature, in positive ionization mode

Peptides in the mucus were characterized using their molecular masses and AAS (Figures 1 and 2). Using MALDI‐MS analyzes the various mucus peptides with an MW below 3 kDa were determined (Figure 1).

The AAS of the peptide [M + H]^+^ at m/z 1039.50 Da, is presented in Figure 2. After tracing the fragment y‐ions and b‐ions of the MALDI‐TOF MS/MS spectrum, the primary structure was determined as LLFSGGQFNG.

The AASs of the most of identified peptides from *C. aspersum* mucus contain predominantly Gly, Leu, Pro, Val, Phe, Ala, Asp, Asn, and Trp residues, typical for many antimicrobial peptides, which are important components of the innate immune system. Most of them have a positive net charge, pI < 7.0, and hydrophobic surface (GRAVY > 0). The mucus contains both cationic and anionic peptides, but cationic are dominant, which are characterized by an amphipathic structure and predominantly hydrophobic surfaces (Dolashki et al., 2020). This fact is considered a prerequisite for their antimicrobial activity—destruction of biological membranes and/or direct cell lysis (Brogden, 2005; Brown & Hancock, 2006; Dolashki et al., 2020; Giuliani et al., 2007).

The results in Table 3 show that several peptides 1, 3, 4, 10, 11, 12, 13, and 14 are predicted to have antibacterial activity, while peptides 1 and 13 might also have antiviral activity. Peptides 10, 12, 13, and 14 were predicted to have both antibacterial and antifungal activity. Peptides with the highest prognostic antibacterial and antifungal activity have high levels of glycine and leucine residues, as well as 1 or 2 proline residues. This indicates they belong to a new class of Gly/Leu‐rich antimicrobial peptides (Dolashki et al., 2018, 2020; Velkova et al., 2018).

The number of the key bacterial groups was determined using the cultivation techniques. These analyses possess good reproducibility, can be performed in every microbiology laboratory and thus give information that is comparable with other studies. These techniques however are too narrow in scope since only 1% of the bacteria in the environment could be cultivated (Sperstad et al., 2011). Cultivation analyses estimate the live bacteria in the studied habitat (the snail mucus). They also give information about the bacterial count from different physiological and taxonomic groups that can grow as pure cultures in the selective and differential media. The determination of the main bacterial groups by cultivation was complemented with isolation of the dominating microbial cultures on nutrient agar and GSP agar.

**Figure 3:**
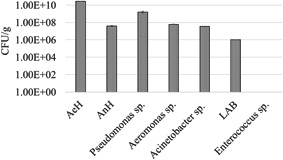
Quantity of the bacterial groups in the mucus studied by cultivation. AeH, aerobic heterotrophs; AnH, anaerobic heterotrophs; LAB, lactic acid bacteria

**Figure 4:**
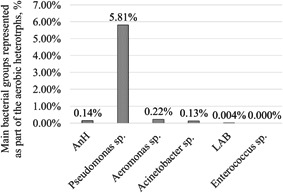
Quantity of the main bacterial groups as part of the aerobic heterotrophs (AnH, anaerobic heterotrophs; LAB, lactic acid bacteria). The quantity of the aerobic heterotrophs was assumed at 100%

The cultivation analyses demonstrated a high number of most of the studied bacteria. The highest quantity was found for the aerobic heterotrophs (2.71 × 10^10^ CFU/g). The number of bacteria with heterotrophic metabolism that can live in the absence of oxygen was also high—3.71 × 10^7^ CFU/g (Figure 3). Among the other bacterial groups which include a specific taxonomic range, the representatives of the genus *Pseudomonas* are most abundant. They represent 5.81% of the aerobic heterotrophs. The quantity of the *Aeromonas* sp. (6.05 × 10^7^ CFU/g) and the LAB bacteria (1.0 × 10^6^ CFU/g) is elevated. The bacteria from the genus *Acinetobacter* are also found in high numbers—3.41 × 10^7^ CFU/g. At the same time, no representatives from the genus *Enterococcus* were recorded in the mucus of *C. aspersum*. The percentage of the aerobic heterotrophs is shown in Figure 4.

The presented results demonstrated that most of the microbial community cannot be cultivated on these most commonly used media. That is why molecular analyses were performed. They do not depend on cultivation and give direct information about the bacteria in the sample.

**Figure 5:**
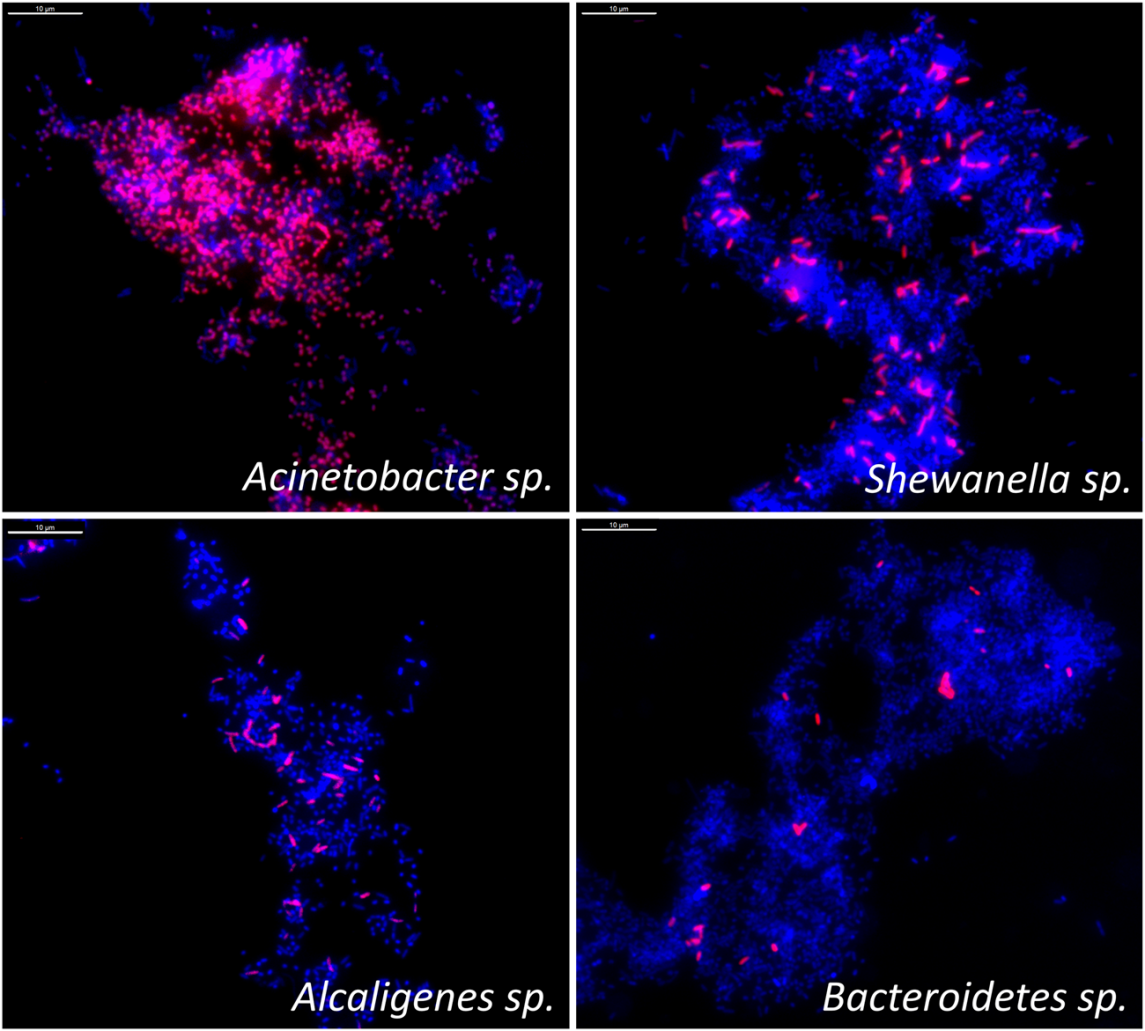
Images from the fluorescence in situ hybridization analysis. The target bacteria are in red. The samples were counterstained with DAPI (in blue). The marker on the pictures is 10 µm

**Figure 6:**
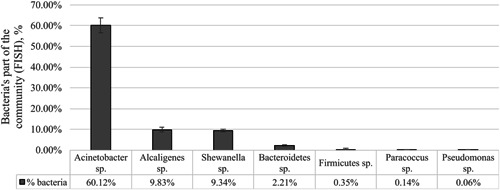
Digital image analysis of the fluorescence in situ hybridization (FISH) results

FISH analysis, performed for some of the most common bacteria in the environment, demonstrated that in the snail mucus *Acinetobacter* sp. prevailed (Figure 5). They were found to be 60% of the community. Representatives from genera *Alcaligenes*, *Shewanella*, and the phylum *Bacteroidetes* were also found in a notable quantity (Figure 6).

From the images, in Figure 5, another feature of the mucus community can be seen. The bacteria from the dominating *Acinetobacter* are found in large clusters whereas the bacteria from the other groups are more or less dispersed in the sample.

The bacteria from the genus *Alcaligenes* were estimated to be almost 10% of the bacteria in the *C. aspersum* mucus (Figure 6).

In the mucus samples analyzed in the present study, the representatives of the genus *Shewanella* were 9% of the bacterial community. This makes them one of the main bacterial groups in it.

The data from the FISH analysis revealed also that the representatives of the *Firmicutes* were only 0.35% from the community. This vast phylogenic group contains many genera, including the LAB bacteria and genus *Enterococcus*. The obtained results confirm the data from the cultivation analyses where these bacteria were found in low numbers.

The bacteria from the genus *Paracoccus* were also included in the FISH analyses as main environmental microorganisms and as ones often found in snail gut samples. In the present study, they were also found in very low quantities in the *C. aspersum* mucus (0.14%, Figure 6). As previously demonstrtaed, these microorganisms are present in the snail gut microbiome in high quantities (Hu et al., 2018; Pinheiro et al., 2015).

According to the results obtained using FISH, the bacteria belonging to *Pseudomonas* were estimated at 0.06%. Based on the cultivation analysis, their share in the community was calculated to be 5.81%. This difference might be related to the inability of the cultivation techniques to retrieve complete information for the complex microbial communities. For habitats such as the snail mucus, this is a strong limitation. It is assumed that a significant part of the bacteria enters into permanent relationships. It is also suggested that these relationships may be the basis for the production of metabolic compounds with beneficial properties.

**Figure 7:**
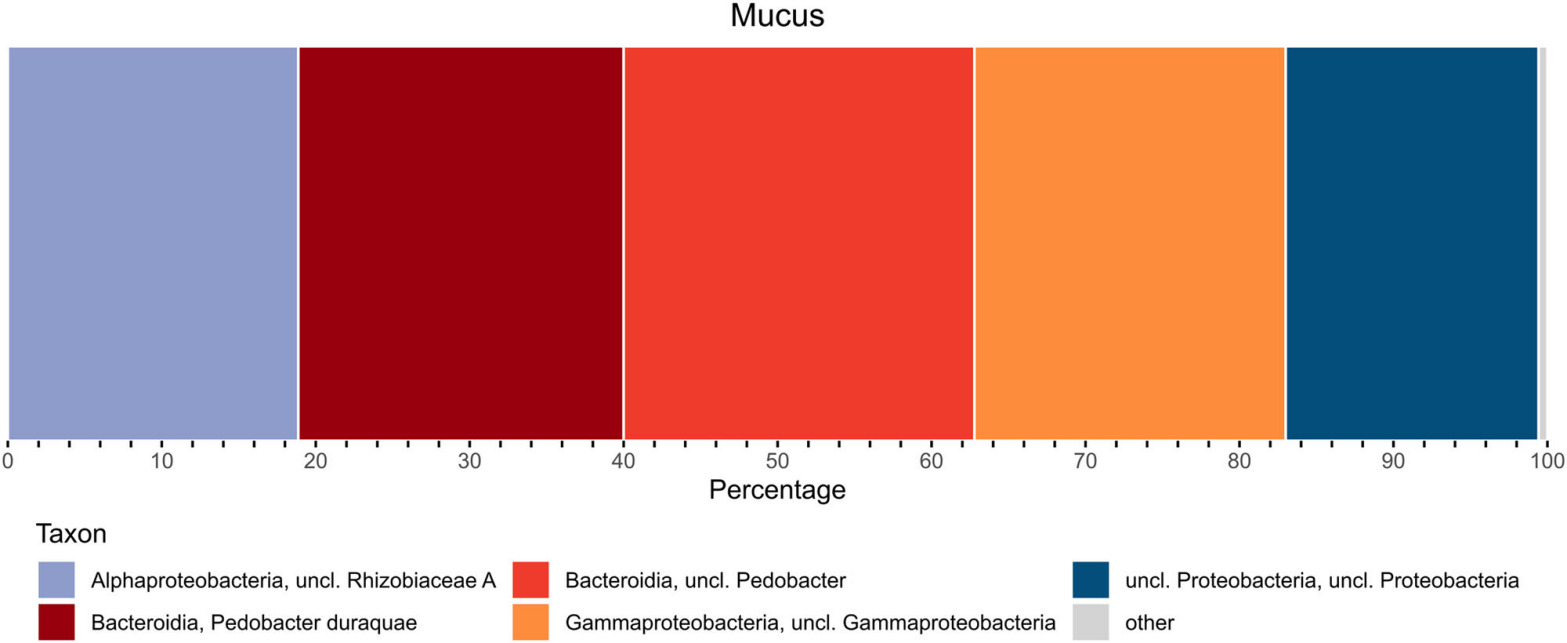
Results from the sequencing of the 16S rRNA gene from the bacterial community in the *Cornu aspersum* mucus

The results from the sequencing analyses demonstrated that most of the community had consisted of three groups of bacteria—*Rhizobiaceae*, *Pedobacter*, and *Gammaproteobacteria* (Figure 7).

Almost one‐fifth of the OTU counts in the samples were identified as *Rhizobiaceae* (19%). These bacteria are usually found in plant‐related communities and were expected to be discovered because of the plant‐based diet of the snails.

Another observation is the high share of the *Pedobacter* representatives in the samples (44% of the OTU counts). Similar information is reported by Pawar et al. (2012) for a microbiome from the giant African snail (*Achatina fulica*). The quantity of these bacteria is estimated to be 12%–17%. In the present study, however, they consisted much more than that (Table 4).

**Table 4:** Percentage of OTU counts obtained from the sequencing of the 16S rRNA gene from snail mucus

Class	Species	Percentage	Taxon
*Alphaproteobacteria*	uncl. *Rhizobiaceae* A	18.87%	*Alphaproteobacteria*, uncl. *Rhizobiaceae* A
*Bacteroidia*	*Pedobacter duraquae*	21.13%	*Bacteroidia*, *Pedobacter duraquae*
*Bacteroidia*	uncl. *Pedobacter*	22.78%	*Bacteroidia*, uncl. *Pedobacter*
*Gammaproteobacteria*	uncl. *Gammaproteobacteria*	20.22%	*Gammaproteobacteria*, uncl. *Gammaproteobacteria*
uncl. *Proteobacteria*	uncl. *Proteobacteria*	16.43%	uncl. *Proteobacteria*, uncl. *Proteobacteria*
Other	Other	0.57%	Other

The elevated share of the *Gammaproteobacteria* (20%) (Table 4), demonstrated by the sequencing analyses, was expected. This result corresponds to the previous findings showing large quantities of bacteria from this taxon: *Acinetobacter*, *Shewanella*, *Aeromonas*, and *Pseudomonas*.

## 4 DISCUSSION

Bioactive compounds, such as proteins, peptides, and glycopeptides, are present in the mucus of many mollusks (Pitt et al., 2015). Recently, many compounds with antimicrobial and antioxidant activity, as well as regenerative properties were detected in the snail mucus (Dolashki et al., 2020; Pitt et al., 2019; Vassilev et al., 2020). Dolashki et al. (2020) found that different peptide and protein fractions from the mucus of garden snail *C. aspersum*, manifest antibacterial activity against Gram negative (*Pseudomonas aureofaciens* and *Escherichia coli*) and Gram positive (*Brevibacillus laterosporus*) bacterial strains as well the anaerobic bacterium *Clostridium perfringens* (Dolashki et al., 2020).

The obtained data demonstrated that the snail mucus is a material very rich in bacteria. Such bacterial abundance is related to symbiotic relationships with their host. They exchange metabolites and mutually regulate their physiological functions. The registered value for the culturable bacterial groups (10^10^ CFU/g) is higher than for those previously found in other snail parts. In samples from the whole body of *C. aspersum*, Simkiss found 0.71 × 10^6^ CFU/g bacteria (Simkiss, 1985). In gut samples from the same snails, Dar and coworkers counted 10^7^ CFU/g bacteria (Dar et al., 2017).

An interesting result from the present study is the lack of *Enterococcus* sp. which were dominating in the gut of the snails according to Charrier et al. (1998). The data indicate that a distinct microbiome is formed in the mucus of *C. aspersum*. This microbial community differs from the microbiomes in other snail parts and probably is regulated by different mechanisms.

Kim et al. (2015) demonstrated the presence of *Aeromonas* with cellulolytic activity in samples from a snail gut. In the present study, the bacteria from this genus were found to be dominating on the nutrient agar and on the GSP medium which could be related to the fact that the snails are herbivores.

According to the FISH results, however, the genus *Acinetobacter* has been found to make up 60% of the bacteria from the community in the mucus. Other authors also identified *Acinetobacter* as the main bacteria in the snail microbiome (Cardoso et al., 2012; Ducklow et al., 1981). Ekperigin found a cellulolytic activity of a culture belonging to the genus *Acinetobacter* (Ekperigin, 2007). The culture was isolated from terrestrial snails and its presence is probably related to the plant‐based food consumed by these animals.

These bacteria were found in large clusters in the mucus (Figure 5). Clustering provides benefits for the microorganisms such as coordinated behavior, the establishment of symbiotic relationships, and the presence of a higher concentration of many metabolites. We can hypothesize that the *Acinetobacter* sp. could have some contribution to the content of the mucus and its specific features described by the mass spectrometric analyses. The clarification of the functional characteristics on the community level, and especially those concerning *Acinetobacter*, will be one of the future directions of our work.

The quantity of the bacteria from the genus *Alcaligenes* which are natural inhabitants of the soil (Batt, 2014), was found to be almost 10% in the *C. aspersum* mucus (Figure 6). Other studies on snail microbiomes did not mention these microorganisms amongst the major bacterial groups. However, the presence of typical soil bacterial groups in the mucus could be explained by the close contact of the snails with the soil surface.

The bacteria from the genus *Shewanella* were also identified as a major part of the snail microbiome (Cardoso et al., 2012; Kim et al., 2015). In the present study, they were estimated to be 9%. Some studies relate these bacteria to the biodegradation of complex polysaccharides in the marine environment (Ito et al., 2019; Li et al., 2016). This was not investigated here but it is a probable reason for their significant share in the mucus microbiome.

The data from the FISH analysis demonstrated a very low share for the large taxonomic group of *Firmicutes* (0.35%). The cultivation analyses confirmed the result—the LAB bacteria were only 0.004% and *Enterococcus* sp. was not found. Both groups belong to the *Firmicutes*. The sequencing also did not provide data for them. At the same time, the studies focused on the terrestrial snail gut suggested the dominant role of *Firmicutes* (Charrier et al., 2006; Nicolai et al., 2015). Those results again highlight the possible differences in microbiomes from distinct snail parts (gut, whole body, mucus).

According to the sequencing results, one of the main bacterial groups in the snail mucus was *Rhizobiaceae*. Representatives of the *Rhizobiaceae* family enter symbiotic relationships with plants. Snails feed on plants and it could be suspected that *Rhizobiaceae* found a suitable niche in the snail mucus.

Sequencing data also demonstrated that the bacteria from genus *Pedobacter* were represented in very high quantities. More than 40% of the OTU counts belonged to this group. This is probably related to the fact that the bacteria from this genus can degrade complex polysaccharides such as heparins. Many of these could be found in the snail mucus (Chinaka et al., 2021; Shaya et al., 2006).

## 5 CONCLUSIONS

The results from the present study on the microbial diversity of the *C. aspersum* mucus, demonstrated that the main bacteria belonged to the genus *Acinetobacter*, *Rhizobiaceae* family, genera *Pedobacter*, *Aeromonas*, *Shewanella*, and *Alcaligenes*. The cultivation analyses, FISH, and the sequencing showed that the microbiome of the mucus possesses unique characteristics. They suggested that the microbial community in the *C. aspersum* mucus differs from those found in other snail parts described in the literature. One of the most distinctive features of it is the prevalence of bacteria known as typical soil and plant‐related bacteria.

Our investigation gives a basis for a hypothesis that these bacteria play a significant role in the formation of the valuable content of the snail mucus. Simultaneously the question “from where this microbiome originates and to what extend its autochthonous or allochthonous nature is key for the features of this valuable resource” remains. Another question that deserves long‐term investigation is what the differences in the snail microbiome in respect to the season, location, and age of the snails are. These details will be a subject of our future investigations. All this will elucidate in greater detail the relation of the microbiome with the isolated and biologically active therapeutic and cosmetic compounds.

## CONFLICT OF INTERESTS

None declared.

## ETHICS STATEMENT

None required.

## AUTHOR CONTRIBUTIONS

**Mihaela Belouhova**: methodology; investigation; writing—original draft preparation, review and editing. **Elmira Daskalova**: investigation. **Ivaylo Yotinov**: investigation. **Yana Topalova**: conceptualization; writing—original draft preparation, review and editing; supervision; project administration; funding acquisition. **Lyudmila Velkova**: methodology; investigation; Aleksander Dolashki: methodology; investigation. **Pavlina Dolashka**: conceptualization; investigation; writing—original draft preparation, review and editing; supervision; project administration; funding acquisition.

## Data Availability

Data are provided in the results section of this paper except for the sequencing data available in the NCBI database under accession number PRJNA790715: https://www.ncbi.nlm.nih.gov/bioproject/PRJNA790715 and the MALDI‐MS/MS peptide data available in figshare at https://doi.org/10.6084/m9.figshare.17879543
